# Clarification of public health pathways toward clinical careers: a pilot study describing a benchmark analysis project

**DOI:** 10.3389/fpubh.2025.1534403

**Published:** 2025-04-23

**Authors:** Elizabeth A. Brown, Melissa Feliciano, Daniel Martinez

**Affiliations:** ^1^Department of Health Behavior, Policy, and Management, Joint School of Public Health at Old Dominion University and Norfolk State University, Old Dominion University, Norfolk, VA, United States; ^2^School of Population & Health Sciences, College of Arts & Sciences, Dillard University, New Orleans, LA, United States

**Keywords:** undergraduate students, public health curricula, basic sciences, clinical careers, pilot study, benchmark analysis

## Abstract

**Introduction:**

Undergraduate public health programs have adapted the required basic sciences based on medical and technological advances and the needs of the public health workforce. We aim to demonstrate how a benchmark analysis project was used to improve curriculum and develop a model where basic sciences in public health can be used to build pathways to clinical graduate programs like medicine, pharmacy, physician assistant, and dental.

**Methods:**

Authors conducted a benchmark analysis of Bachelor of Science in Public Health (BSPH) Degrees (or public health-related degrees) in the southern region of the United States (US) to explore required basic sciences. Authors searched the Council on Education for Public Health’s website to identify accredited, baccalaureate public-health related degrees. The US Census Bureau’s Geographic Levels was used to define the southern region of the US. Inclusion criteria for public health-related programs included the following characteristics: (1) accredited as of October 2023, (2) located in the southern region of the US, and (3) Bachelor of Science. Authors used the American Medical College Application Service Application Course Classification Guide to categorize basic sciences into three categories: Biology, Chemistry, and Physics. Data were extracted to an Excel document for further review and analysis.

**Results:**

There were 38 accredited, baccalaureate public health-related programs in the southern region of the US that met the inclusion criteria. Eighteen programs (47.4%) had at least one concentration or track. Most programs required General or Introductory Biology (*n* = 12), Anatomy & Physiology (*n* = 9), General or Introductory Chemistry, or Microbiology (*n* = 5). Physics was counted twice as a required course while Cellular Biology (or Molecular Biology) and Organic Chemistry were counted once each across 38 programs.

**Discussion:**

There are substantial differences across required basic sciences for BSPH Degrees in the southern region of the US. We offer a model of required basic sciences for BSPH leadership and students to consider adopting as they market BSPH Degrees for students interested in clinical careers and for those students not interested in clinical professions. This project and proposed model demonstrate that a BSPH Degree is a viable route to clinical careers, depending on a student’s academic and professional interests.

## 1 Introduction

Public health is an extremely broad subject, and as a result, undergraduate public health curricula and education can span an array of topics and issues to prepare students for the public health workforce. For example, public health curricula may focus on topics such as aging, health equity, health policy, injury and violence prevention, mental health, social determinants of health, tobacco, and many other areas that affect community health (1). The public health workforce includes individuals who may work in epidemiology and surveillance, program planning and evaluation, policy and regulatory practices, healthcare administration and services, environmental justice, and disaster planning to name a few (2). Literature over the past few years points to a shortage of public health professionals in local, state, and federal government (2, 3). According to authors, policy decisions that provide funding for public health education, increase capacity for public health education, and improve the diversity of the public health workforce may address shortage challenges (2). One approach to help students discover public health is to embed an introductory public health course into the general education requirements; undergraduates should have an opportunity to access public health education to improve the health of communities and society through public health knowledge, skills and values (4, 5). Another approach to increase the public health workforce is to identify potential barriers in undergraduate public health curricula (e.g., degree type, required courses, etc.). Some undergraduate public health requirements (e.g., basic sciences, internship hours, etc.) may deter students from joining programs or successfully completing the degree, which negatively impacts recruitment and retention. A closer look at undergraduate Public Health Degrees is warranted to identify issues and offer solutions.

Variation across public health curricula for Bachelor of Science and Bachelor of Arts shows some public health programs offer significantly more credit hours of public health-related content (6). The growth of the public health field has led to substantial changes in public health curricula and strategies to focus on quality instruction in accredited undergraduate public health-related programs (7, 8). In a 2018 study, authors found that 95% of public health degrees were a Bachelor of Science; further, from 2003–2016, many of the degrees conferred were in public health education and promotion, general public health, and community health and preventive medicine (7). While there has been much progress toward preparing undergraduate students for their next steps in public health education, a clear model for required science or math courses, with the focus of clinical graduate programs, across public health programs in the United States (US) has yet to be developed. Stakeholders in the public health field must collaborate to decide what science courses an undergraduate student needs to be an ideal candidate for the public health workforce and/or clinical graduate programs.

The Core Competencies for Public Health Professionals and the Council on Education for Public Health (CEPH), alongside the Association of Schools and Programs of Public Health (ASPPH), guide public health education for professionals and students, respectively. Core Competences for Public Health Professionals focus on eight domains from data analytics, program planning, health equity, communication, leadership and systems thinking, and several others (9). These core competencies help link academia, specifically public health education, to public health practice for those who will work in public health roles after graduation. CEPH offers accreditation standards for schools and programs of public health who seek to demonstrate that minimal public health education standards are met. CEPH has 11 foundational domains that advance undergraduate public health education, including concepts and applications of basic statistics, history of public health, social determinants of health in diverse communities, ethical considerations, and public health communication (10). In the sciences, CEPH focuses on foundations of biological and life sciences and disease and health promotion throughout the life cycle (10). Last, CEPH requires students demonstrate two competencies related to communicating, using, and evaluating public health information in both oral and written form (10). These public health competencies offer an excellent foundation for public health programs and educators to ensure students are prepared for the public health workforce in a variety of industries and organizations.

It should be noted that over the past 100 years, public health programs and curricula in the US have changed substantially. In 1918, John Hopkins University School of Hygiene and Public Health became the first endowed school with a dedicated public health program; earlier public health education focused on laboratory sciences and infectious diseases (11). While most universities preferred graduate-level public health programs (7), by 1926, three universities, Massachusetts Institute of Technology (Bachelor of Science), University of Minnesota (Bachelor of Science), and University of California (Bachelor of Arts), offered undergraduate programs in public health (12). These Bachelor of Public Health Programs each required a variety of science coursework where Anatomy, Bacteriology, Chemistry, Physics, Physiology, and Zoology were common across the three programs (Table 1) (12).

**Table 1:** Required basic sciences for the first baccalaureate Public Health Programs in the United States^1^.

Required sciences	Massachusetts Institute of Technology Bachelor of Science	University of Minnesota Bachelor of Science	University of California Bachelor of Arts
Anatomy	X	X	X
Bacteriology	X	X	X
Biochemistry	X		X
Biology - Animal		X	
Biology - General	X		
Botany	X		
Chemistry (General, analytical, organic or inorganic, physical)	X	X	X
Histology	X		
Industrial Microbiology/Microbiology	X		X
Parasitology	X		X
Pathology		X	
Physics (Mechanics, light, heat, electricity)	X	X	X
Physiology	X	X	X
Zoology	X	X	X

^1Green highlight implies all three programs required the science, yellow highlight indicates only two programs required the science, and red highlight indicates only one program required the science.^

The required science coursework ensured undergraduate students had the scientific knowledge of disease processes to reduce disease incidence and prevalence in the early 20^th^ century. Today, undergraduate students take a wide array of required sciences in public health programs nationwide.

Through accreditation standards, market analyses, and faculty and student feedback, programs can gain invaluable insight into modifications needed to ensure students are competitive in the public health workforce. Currently, students pursuing a Bachelor of Science in Public Health (BSPH) or public health-related degrees typically pursue one of four key pathways after graduation: (1) graduate clinical programs, (2) Master’s in Public Health, (3) entry-level, public health jobs, and/or (4) additional work-related experience (e.g., global public health with Peace Corps) (13). Further, conducting a market analysis of typical career paths in the local sector for BSPH students provides valuable insight into the skills students will be expected to have upon graduation (14). While electives can be utilized to help students pursue their specific interests (e.g., medical school, physician assistant program, pharmacy, etc.), it is essential that students have a robust knowledge of various foundational public health skills and competencies they can build on after graduation (13).

One approach to improving undergraduate public health curricula is reviewing required courses, especially science and math courses, across accredited undergraduate public health programs and creating a model aligned with various paths for public health students. A recent study conducted a narrative review of CEPH-accredited bachelor programs in public health; however, authors excluded required non-public health courses and general education courses, including basic sciences (e.g., chemistry, physics, and other science courses) (15). There is variability in public health curricula. To our knowledge, there is no benchmark analysis that (1) reviews required basic sciences of accredited public health-related undergraduate programs or (2) publishes a model of basic sciences to promote pathways to clinical graduate programs across undergraduate public health programs in the US that connect to CEPH’s March 2024 Accreditation Criteria for Public Health Bachelor’s Degree Foundational Domains (Domain #2: Foundations of biological and life sciences) and (Domain # 6: Underlying science of human health and disease).

The purpose of this benchmarking project was to (1) examine accredited, Bachelor of Science public health-related programs in the southern region of the US; (2) describe required science courses that align with CEPH’s Foundational Domain #2 and Domain #6; (3) advocate for modifying required basic science (also referred to as bench sciences) courses to promote flexibility, access, and inclusivity based on different students’ needs, strengths, and abilities; and (4) propose a model for basic sciences, creating pathways to popular clinical-based graduate programs, which may all support public health programs’ recruitment and retention efforts.

## 2 Methods

Authors searched CEPH’s website (16) to identify accredited, baccalaureate public health-related programs in the southern region of the US. The team reviewed public health-related programs in all three groups on CEPH’s list: (1) schools of public health, (2) public health programs, and (3) standalone baccalaureate programs. Only public health-related programs in the southern region of the US, as defined by the US Census Bureau’s Geographic Levels (17), were reviewed. The South Atlantic Division included the following states: Washington, DC, Delaware, Florida, Georgia, Maryland, North Carolina, South Carolina, Virginia, and West Virginia. The East South Central Division included the following states: Alabama, Kentucky, Mississippi, and Tennessee. The West South Central Division included the following states: Arkansas, Louisiana, Oklahoma, and Texas.

Inclusion criteria for public health-related programs included the following characteristics: (1) accredited as of October 2023, (2) located in the southern region of the US, and (3) Bachelor of Science. For public health programs, if the college or university had a baccalaureate public health-related program (e.g., Health Promotion, Public Health Studies, etc.), we included that data. We excluded colleges and universities with public health programs that specifically identified as a Master of Public Health (MPH) Program or doctoral program in public health. Program/degree names that did not specifically state “science” (e.g., Bachelor of Public Health) were excluded. Where possible, researchers used the link provided by CEPH to locate school/program information, specifically required sciences and concentrations.

Authors entered all relevant data in a cloud-based Excel document so that authors could work on the document in real time. Each division (South Atlantic, East South Central, and West South Central) had its own tab in the Excel file. Each tab had the following columns for data entry/organization: Institution name and state, program name, concentrations, required science courses (and credits), required math courses (and credits), additional notes, and program website. Another Excel document was created to count specific science requirements across the programs as the initial Excel document included a substantial amount of information.

We used the American Medical College Application Service (AMCAS) Application Course Classification Guide to categorize sciences: Biology (Anatomy and Physiology, Biology, Genetics, Cell Biology, Genetics, Microbiology, etc.), Chemistry (Biochemistry, Chemistry, Toxicology, etc.), and Physics (Astronomy or Physics) (18). To simplify count data associated with sciences, we combined Molecular Biology with Cellular Biology, and we combined Physiology with Anatomy. Last, we added Organic Chemistry to the AMCAS Chemistry category. When classifying required sciences, we only used data that specifically listed the required science’s entire name (Biology, Chemistry, Genetics, etc.) instead of the course prefix (e.g., BIOL, CHEM, ENVS, etc.) because BIOL could mean any biological science (e.g., Genetics, Cellular Biology, etc.). Some programs provided a list of sciences or “natural sciences” where students could select a specific number of credit hours from several sciences listed by prefix, so we excluded these as well. Some programs listed different required sciences based on the concentration or track. We chose not to count the required sciences by concentration in the final analysis to promote generalizability across the undergraduate public health degree, not necessarily the concentration or track (e.g., pre-clinical track). Last, we could not locate any required science information for only one program.

Two authors reviewed US & News World Report (19) or Scimago (20) for top graduate programs in specific fields and national organizations—Association of American Medical College or AAMC (21), American Association of Colleges of Pharmacy or AACP (22), and American Dental Education Association or ADEA (23)—and then visited the admissions page of the top three programs in the US to ensure our suggested course list matched prerequisites for top programs in the nation. The final analysis for the benchmark analysis occurred in March 2024, and at least two authors reviewed extracted data in fall 2023 and then again in fall 2024 to ensure accuracy.

## 3 Results

There were 38 accredited, baccalaureate public health-related programs in the southern region of the US that met the inclusion criteria: South Atlantic Division (22 programs), East South Central Division (10 programs), and West South Central Division (6 programs). There were 16 within Schools of Public Health, 13 within public health programs, and 9 stand-alone programs. Most program names were Bachelor of Science in Public Health (B.S. Public Health). Some program names included Health Science, Community Health, Community Health Education, Health Promotion, and Public Health Studies.

### 3.1 Concentrations and tracks

Eighteen programs (47.4%) had at least one concentration or track. Most concentrations were in Community Health Education and Promotion (*n* = 9) or Pre-Medical/Clinical Science/Pre-Health (*n* = 5). Other concentrations or tracks included Health Policy & Management or Health Administration, Environmental Health, Dietetics and Nutrition, and Genetics.

### 3.2 Required science courses

For the Biology category, programs required General or Introductory Biology (*n* = 12), Anatomy & Physiology (*n* = 9), or Microbiology (*n* = 5), where most did not specify a required lab to accompany the lecture. Only one program required Cellular Biology or Molecular Biology, and only one program required Genetics. For the Chemistry category, programs required General or Introductory Chemistry (*n* = 8). Only one program required Organic Chemistry. For the Physics category, two programs required a Physics course. Programs where we could not count or ascertain the type of science (e.g., introductory vs. general) were most likely to have concentrations or tracks.

### 3.3 Suggested basic science courses

Based on findings from this benchmark analysis, our program adopted a preliminary model of required basic sciences, removing previously required basic sciences like Organic Chemistry, Physics, and Genetics and allowing lower-level introductory basic sciences to promote access and inclusivity for diverse student strengths and abilities (Table 2).

**Table 2:** Model of basic sciences based on benchmark analysis^1^.

Bachelor of Science in Public Health - required science courses	
Science courses	Credit hours
Introductory Biology I + Lab ~*or~* General Biology I + Lab	4
Introductory Biology II + Lab *~or~* General Biology II + Lab	4
Introductory Chemistry I + Lab *~or~* General Chemistry I + Lab	4
Introductory Chemistry II + Lab *~or~* General Chemistry II + Lab	4
Human Anatomy & Human Physiology I + Lab	4
Human Anatomy & Human Physiology II + Lab	4
Total credit hours toward BSPH Degree	24

^1The goal is to allow flexibility for an introductory biology or chemistry course sequence versus an advanced biology or chemistry course sequence based on the students’ academic and professional goals.^

#### 3.3.1 Suggested basic science courses for clinical programs

Additionally, authors offer suggested science courses for public health students who are interested in applying to graduate programs, such as medicine, physician assistant, pharmacy, dentistry, etc. (Tables 3–6).

**Table 3:** Suggested basic sciences for Medical School^1,2^.

Bachelor of Science in Public Health - required science courses	
Science courses	Credit hours
Introductory Biology I + Lab ~*or~* General Biology I + Lab	4
Introductory Biology II + Lab *~or~* General Biology II + Lab	4
Introductory Chemistry I + Lab *~or~* General Chemistry I + Lab	4
Introductory Chemistry II + Lab *~or~* General Chemistry II + Lab	4
Human Anatomy & Human Physiology I + Lab	4
Human Anatomy & Human Physiology II + Lab	4
Credit hours toward BSPH Degree	24
*Suggested* science courses for Medical School	
Organic Chemistry I + Lab	4
Organic Chemistry II + Lab	4
Biochemistry	3
Physics I + Lab	4
Physics II + Lab	4
Genetics	3
Cellular Biology ~or~ Molecular Biology	3
Introductory Microbiology + Lab	4
Credit hours toward Medical School	29
	53 total credit hours

^1Each graduate program has specific prerequisites, and some programs may even have specific time periods for completion of prerequisites. Students should work with their academic advisor to review prerequisites. Students are also encouraged to contact the Admissions Office for the graduate program of interest to verify required prerequisite courses.^

^2^In August 2024, we reviewed both the Association of American Medical College or AAMC website and the three top programs in the United States (based on US News & World Report): Baylor College of Medicine, Case Western Reserve University School of Medicine, and Emory University School of Medicine.

**Table 4:** Suggested basic sciences for Physician Assistant Programs^1,2^.

Bachelor of Science in Public Health - required science courses	
Science courses	Credit hours
Introductory Biology I + Lab ~*or~* General Biology I + Lab	4
Introductory Biology II + Lab *~or~* General Biology II + Lab	4
Introductory Chemistry I + Lab *~or~* General Chemistry I + Lab	4
Introductory Chemistry II + Lab *~or~* General Chemistry II + Lab	4
Human Anatomy & Human Physiology I + Lab	4
Human Anatomy & Human Physiology II + Lab	4
Credit hours toward BSPH Degree	24
*Suggested* science courses for Physician Assistant Program	
Organic Chemistry I + Lab	4
Organic Chemistry II + Lab	4
Biochemistry	3
Genetics	3
Cellular Biology ~or~ Molecular Biology	3
Introductory Microbiology + Lab	4
Credit hours toward Physician Assistant Program	21
	45 total credit hours

^1Each graduate program has specific prerequisites, and some programs may even have specific time periods for completion of prerequisites. Students should work with their academic advisor to review prerequisites. Students are also encouraged to contact the Admissions Office for the graduate program of interest to verify required prerequisite courses.^

^2^In February 2025, we reviewed both the American Academy of Physician Associates (AAPA) website and the three top programs in the United States (based on US News & World Report): Duke University School of Medicine’s Physician Assistant Program, Baylor College of Medicine’s Physician Assistant Program, and University of Iowa’s Carver College of Medicine’s Physician Assistant Program.

**Table 5:** Suggested basic sciences for Pharmacy Programs^1,2^.

Bachelor of Science in Public Health - required science courses	
Science courses	Credit hours
Introductory Biology I + Lab ~*or~* General Biology I + Lab	4
Introductory Biology II + Lab *~or~* General Biology II + Lab	4
Introductory Chemistry I + Lab *~or~* General Chemistry I + Lab	4
Introductory Chemistry II + Lab *~or~* General Chemistry II + Lab	4
Human Anatomy & Human Physiology I + Lab	4
Human Anatomy & Human Physiology II + Lab	4
Credit Hours toward BSPH Degree	24
*Suggested* science courses for Pharmacy Program	
Organic Chemistry I + Lab	4
Organic Chemistry II + Lab	4
Physics I + Lab	4
Biochemistry	3
Genetics	3
Introductory Microbiology + Lab	4
Credit hours toward Pharmacy Program	22
	46 total credit hours

^1Each graduate program has specific prerequisites, and some programs may even have specific time periods for completion of prerequisites. Students should work with their academic advisor to review prerequisites. Students are also encouraged to contact the Admissions Office for the graduate program of interest to verify required prerequisite courses.^

^2^In February 2025, we reviewed both the American Association of Colleges of Pharmacy or AACP website and the three top programs in the United States (based on US News & World Report): University of North Carolina-Chapel Hill, University of California-San Francisco, and University of Michigan-Ann Arbor.

**Table 6:** Suggested basic sciences for Dental Programs^1,2^.

Bachelor of Science in Public Health - required science courses	
Science courses	Credit hours
Introductory Biology I + Lab ~*or~* General Biology I + Lab	4
Introductory Biology II + Lab *~or~* General Biology II + Lab	4
Introductory Chemistry I + Lab *~or~* General Chemistry I + Lab	4
Introductory Chemistry II + Lab *~or~* General Chemistry II + Lab	4
Human Anatomy & Human Physiology I + Lab	4
Human Anatomy & Human Physiology II + Lab	4
Credit Hours toward BSPH Degree	24
*Suggested* science courses for Dental Program	
Organic Chemistry I + Lab	4
Organic Chemistry II + Lab	4
Physics I + Lab	4
Physics II + Lab	4
**Biochemistry (recommended)	3
**Microbiology (recommended)	3
**Cell Biology (recommended)	3
Credit Hours toward Dentistry Program	25
	49 total credit hours

^1Each graduate program has specific prerequisites, and some programs may even have specific time periods for completion of prerequisites. Students should work with their academic advisor to review prerequisites. Students are also encouraged to contact the Admissions Office for the graduate program of interest to verify required prerequisite courses.^

^2^In August 2024, we reviewed both the American Dental Education Association or ADEA website and the three top programs in the United States (based on Scimago Institutions Rankings): University of Michigan (Doctor of Dental Surgery or DDS), Harvard University (Doctorate of Dental Medicine or DDM), and University of Washington (DDS Program).

## 4 Discussion

Our findings highlight a varied offering of required sciences across 38 baccalaureate public health programs in the southern US. Most programs lean toward Introductory or General Biology, Introductory or General Chemistry, and Anatomy and Physiology for their required sciences; however, due to the variation, we saw that some programs required Microbiology, Genetics, Physics, and Organic Chemistry. Our study describes a benchmark analysis project where we used results to improve curriculum, increase flexibility for students, and align with CEPH’s March 2024 Accreditation Criteria for Public Health Bachelor’s Degree Foundational Domains (Domain #2: Foundations of biological and life sciences) and (Domain #6: Underlying science of human health and disease). Further, for those students who are interested in clinical professions, we offer a list of suggested science courses for students to apply to in-demand, clinical, graduate programs. The goal of this project is three-fold: (1) conduct a benchmark analysis project to inform curriculum changes, (2) create a model to market BSPH Degrees as ideal pathways to clinical graduate programs, and (3) give students the choice to take more advanced sciences based on their individualized public health career goals in hopes of supporting public health programs’ recruitment and retention efforts. Flexibility and autonomy regarding basic sciences may be a creative way to market public health degrees, especially as we face challenges recruiting students.

While there are public health organizations that focus on quality education and training in various ways to strengthen the next generation of public health professionals (24), to our knowledge, we are not aware of a benchmark analysis to inform a model of required basic sciences, specifically for baccalaureate public health programs in the US, that promote pathways to clinical graduate programs. CEPH, recognized by the U.S. Department of Education, offers guidance on building quality instruction and accredits public health programs and schools after in-depth examination (8). CEPH requires that bachelor programs address several Public Health Domains (e.g., math/quantitative reasoning, human health, science, etc.). For the Science Public Health Domain, CEPH requires an accredited program or applicant program to provide evidence that the program addresses biological, life, and human health and disease sciences (15). Additionally, BSPH programs must demonstrate they have two competencies—public health communication and information literacy—addressed within the program’s required courses and assignments (15). Last, ASPPH offers resources to support innovative education, research, and practice to public health leaders, students, and faculty (25).

Individuals may consider several certifications to demonstrate expertise in public health based on their preferences and future career goals; however, there is no explanation or justification for requiring excessive advanced basic sciences in undergraduate public health programs. Several certifications include the National Commission for Health Education Credentialing’s (NCHEC) Certified Health Education Specialist (CHES) or Masters Certified Health Education Specialist (MCHES) to highlight expertise or competency in health education (26) and (2) National Board of Public Health Examiners’ (NBPHE) Certified in Public Health (CPH) Exam (27). The NCHEC CHES and MCHES exams focus on Eight Areas of Responsibility—Needs Assessment, Planning, Implementation, Evaluation, and others—but do not require a specific focus on sciences (28). The NBPHE CPH Exam focuses on key public health areas, including data analysis and informatics, communication, disease prevention and injury reduction, and others (29); further, there is a domain for public health biology and human disease risk (24). Public health students do not necessarily need expertise or courses in specific sciences (e.g., Botany, Genetics, Organic Chemistry, Microbiology, Physics, Toxicology, etc.) to successfully complete these examinations; however, these advanced basic sciences could prove to be advantageous to those students interested in pursuing clinical graduate degrees. It is critical to note that all public health students may not need advanced basic science courses based on their academic strengths and weaknesses, their professional career goals, and their interests.

There is an opportunity to create an approach that can potentially increase college admissions, support program leadership, promote more options for public health students, and even address shortages in healthcare fields. In the early 1900s, there was an emphasis on basic sciences and a push to have public health complement medicine (24). However, today, we can create a vision and develop a model where the advanced basic sciences are not required for undergraduate public health students but are suggested as a subset of public health for students who want to pursue a clinical or graduate degree that requires specific science coursework. As colleges and universities face the anticipated enrollment cliff of 2025, they need to provide access to not only a college education but also a logical pathway to potential graduate programs, particularly those healthcare careers facing shortages. According to the Health Resources & Services Administration (HRSA) Health Workforce Projections, many health fields will face shortages, including those in allied health (opticians, physical therapists, and pharmacists), behavioral health (addiction counselors and mental health counselors), nursing (registered nurses and licenses practical nurses), oral health (dental hygienists and general dentists), and medicine (primary care physicians, cardiology physicians, and OB-GYNs) (30). Authors from a recent study suggest undergraduate public health programs offer ideal opportunities for students to complete pre-med and science courses (15). Outside of these potential benefits for pathway programs, stakeholders, especially policymakers, will need to advocate for higher education policy changes and funding to help students pay for college or reduce college debt, incentivize students to work with vulnerable populations (e.g., rural communities), and create paid job opportunities while in school.

While our study did not include a review of the social sciences like Psychology, History, or Sociology, these sciences deserve attention as well. Psychology, or the study of human behavior (31), is critical to public health education as public health professionals work with individuals and groups to stop smoking, eat healthy, get timely vaccinations and screenings, and practice safe sexual behaviors. It is important for public health professionals to understand the connection between health behaviors and overall health. Sociology, or the study of social structures and how individuals interact in those systems (32), has a relevant connection to public health. Public health practitioners should have the skills to investigate the relationship between social identities and social determinants of health (e.g., race, gender, social class, religion, etc.) and health equity, especially in diverse communities. It is imperative to have a clear understanding of how social constructs like race, and subsequent behavior of racism, can impact health from a sociology and public health lens. Last, public health students and practitioners should have a well-rounded understanding of American government and policymaking, which can be learned through political science courses. According to the American Political Science Association (APSA), political science consists of studying political ideologies, behaviors, and institutions (33). Government and policies impact health. In fact, it might be safe to say that “Policy is Public Health.” The World Health Organization’s (WHO) Conceptual Framework for the Action on the Social Determinants of Health (CSDH) actually identifies governance and various policies as structural determinants of health inequities (34). Public health organizations and educators understand the importance and relevance of the social sciences and should continue to weave these sciences into public health curricula.

Our study is not without limitations. We did not review all accredited BSPH Degrees (or public health-related programs) across the US; however, we reviewed a substantial number of accredited programs in the US, and we had a variety of programs that were housed in either a school of public health, public health programs, or as a stand-alone program. Therefore, we offer a model for required sciences in BSPH Degrees that we plan to continue vetting with program directors nationwide. We did not review science requirements for Behavioral & Social Sciences (e.g., Psychology or Sociology); however, this was outside the scope of our study. We omitted Bachelor of Arts in Public Health Degrees and focused on Bachelor of Science in Public Health since BSPH Degrees may promote substantial content in science, mathematics, healthcare, and technology. There is a chance we may have missed information when reviewing required sciences in each program; however, at least two authors checked school information at least two times (fall 2023 and fall 2024).

## 5 Conclusion

Our inquiry highlights the need for consistency in marketing BSPH Degrees, required basic sciences, and pathways to clinical graduate programs for students. Based on the data, we recommend public health leadership in academia discuss the proposed model and consider adopting it for their programs for several reasons. This proposed model allows BSPH Degrees to potentially remove some of the required advanced basic sciences and even accept or substitute Introductory Biology or Chemistry courses based on students’ career goals, increasing flexibility and autonomy, which also may help with recruitment and retention in BSPH Degrees. For example, students have different strengths and weaknesses, and students should not face barriers to completing a BSPH Degree because they are not strong in advanced basic sciences. Public health leaders in academia should also consider the benefits of offering a Bachelor of Arts in Public Health, the purpose, and what core courses should be offered for a Bachelor of Arts degree. Our next steps include surveying undergraduate public health program directors, students, and graduate school admissions counselors to get additional feedback concerning the proposed model and suggested courses for different pathways to medicine, pharmacy, etc. We hope to add more suggested science pathways for other programs like occupational therapy, physical therapy, and nursing. It is our goal to be intentional (and offer other programs flexibility) with proposing this model for required basic sciences in undergraduate public health programs that offer pathways to clinical graduate programs, autonomy, and more options to undergraduate public health students who have diverse career interests that may (or may not) require advanced basic sciences for their future career endeavors in public health.

## Data Availability

The raw data supporting the conclusions of this article will be made available by the authors, without undue reservation.
